# Diffusing alpha-emitters radiation therapy in combination with temozolomide or bevacizumab in human glioblastoma multiforme xenografts

**DOI:** 10.3389/fonc.2022.888100

**Published:** 2022-09-27

**Authors:** Yossi Nishri, Maayan Vatarescu, Ishai Luz, Lior Epstein, Mirta Dumančić, Sara Del Mare, Amit Shai, Michael Schmidt, Lisa Deutsch, Robert B. Den, Itzhak Kelson, Yona Keisari, Lior Arazi, Tomer Cooks, Vered Domankevich

**Affiliations:** ^1^ Translational Research Laboratory, Alpha Tau Medical, Jerusalem, Israel; ^2^ The Shraga Segal Department of Microbiology, Immunology and Genetics, Faculty of Health Sciences, Ben-Gurion University, Beer-Sheva, Israel; ^3^ Unit of Nuclear Engineering, Faculty of Engineering Sciences, Ben-Gurion University of the Negev, Beer-Sheva, Israel; ^4^ Radiation Protection Department, Soreq Nuclear Research Center, Yavne, Israel; ^5^ Department of Biomedical Engineering, Faculty of Engineering, Tel Aviv University, Tel Aviv, Israel; ^6^ Physics Laboratory, Alpha Tau Medical, Jerusalem, Israel; ^7^ Biostatistics Department, BioStats Statistical Consulting Ltd., Maccabim, Israel; ^8^ Department of Radiation Oncology, Urology, and Cancer Biology, Thomas Jefferson University, Philadelphia, PA, United States; ^9^ School of Physics and Astronomy, Raymond and Beverly Sackler Faculty of Exact Sciences, Tel Aviv University, Tel Aviv, Israel; ^10^ Department of Clinical Microbiology and Immunology, Sackler Faculty of Medicine, Tel Aviv University, Tel Aviv, Israel

**Keywords:** radiotheapy, alpha particle, antiangiogeic therapy, glioblasoma multiforme, alpha DaRT

## Abstract

Glioblastoma multiforme (GBM) is at present an incurable disease with a 5-year survival rate of 5.5%, despite improvements in treatment modalities such as surgery, radiation therapy, chemotherapy [e.g., temozolomide (TMZ)], and targeted therapy [e.g., the antiangiogenic agent bevacizumab (BEV)]. Diffusing alpha-emitters radiation therapy (DaRT) is a new modality that employs radium-224-loaded seeds that disperse alpha-emitting atoms inside the tumor. This treatment was shown to be effective in mice bearing human-derived GBM tumors. Here, the effect of DaRT in combination with standard-of-care therapies such as TMZ or BEV was investigated. In a viability assay, the combination of alpha radiation with TMZ doubled the cytotoxic effect of each of the treatments alone in U87 cultured cells. A colony formation assay demonstrated that the surviving fraction of U87 cells treated by TMZ in combination with alpha irradiation was lower than was achieved by alpha- or x-ray irradiation as monotherapies, or by x-ray combined with TMZ. The treatment of U87-bearing mice with DaRT and TMZ delayed tumor development more than the monotherapies. Unlike other radiation types, alpha radiation did not increase VEGF secretion from U87 cells in culture. BEV treatment introduced several days after DaRT implantation improved tumor control, compared to BEV or DaRT as monotherapies. The combination was also shown to be superior when starting BEV administration prior to DaRT implantation in large tumors relative to the seed size. BEV induced a decrease in CD31 staining under DaRT treatment, increased the diffusive spread of ^224^Ra progeny atoms in the tumor tissue, and decreased their clearance from the tumor through the blood. Taken together, the combinations of DaRT with standard-of-care chemotherapy or antiangiogenic therapy are promising approaches, which may improve the treatment of GBM patients.

## Introduction

Glioblastoma multiforme (GBM) is the most common and aggressive malignant primary brain tumor (1). The standard treatment for newly diagnosed GBM patients includes surgical resection, followed by radiation therapy and chemotherapy using temozolomide (TMZ) (2, 3). Despite improvement in overall survival offered by this regime, the prognosis of GBM patients remains poor (4) and virtually all GBM tumors relapse (5).

Various strategies are investigated for treating primary and recurrent GBM patients. One approach involves targeted therapy with the humanized vascular endothelial growth factor (VEGF) monoclonal antibody bevacizumab (BEV), which is already approved for clinical use for recurrent GBM (3). Glioma cells are a major source of VEGF (6), a key mediator of angiogenesis (7) that correlates with malignancy grade and poor prognosis (6). The use of BEV disrupts angiogenesis. When employed in combination with chemotherapy and radiation therapy, this can improve the tumor response by reducing hypoxia and assisting drug delivery, which is otherwise precluded by the disorganized architecture of the tumor vascular system (6). This is especially important in GBM, where—as a baseline—the blood–brain barrier (BBB) in itself reduces drug delivery dramatically (e.g., only 30% of plasma TMZ passes the BBB) (8). BEV has indeed been shown to improve progression-free survival and performance status in patients with GBM, but this does not translate into an overall survival benefit (9, 10). It was suggested that treatment by BEV initially decreases tumor hypoxia and reduces tumor edema, during a transient normalization phase (6), but that this effect is followed by a reduction in vessel density and blood flow (11), which induces a metabolic switch towards anaerobic glycolysis (6) and increased cell invasion (11). Notably, VEGF was reported to be upregulated upon low linear energy transfer (LET) irradiation (12–14), as demonstrated in different glioma cell lines and human xenografts (6), and the involvement of transactivating factors, such as HIF-1, was suggested (15). Therefore, low-LET radiation therapies may disturb BEV function by leading to an excess of VEGF relative to the amount of available VEGF antibodies. Little is known about the effect of high-LET radiation on VEGF secretion.

The combination of TMZ with different qualities of radiation was previously investigated in U87 cells, showing that the relative biological effectiveness (RBEs) of the combined treatment increases with LET. The DNA damage induced by alpha particles was more severe than by x-rays or protons, as evidenced by a slower rate of disappearance of DNA damage foci after irradiation (16). Alpha particles are widely known to have a high cytotoxic effect (17), which is largely insensitive to the oxygenation state of the cell (18), and is mediated by the creation of clustered, difficult-to-repair, double-strand breaks (DSBs) through direct ionization (19, 20). The short range of alpha particles (~40–90 μm in tissue) can further minimize collateral damage if an effective delivery scheme is used to bring the alpha-emitting atoms to the immediate vicinity of the cancer cells.

Recent advances in nuclear medicine opened novel avenues for alpha particle-based treatments (21–23). These employ antibodies, peptides, small molecules, and nano- or micro-particles carrying alpha-emitting atoms, which are injected either locally or systemically and target cancer cells. Recently, efforts in this field have also been directed towards recurrent GBM management (24–26). Targeted alpha therapy (TAT) using molecular vectors labeled by ^213^Bi, ^211^At, and ^225^Ac, delivered by intracavity or intratumoral injection, have proven to be safe and well-tolerated by GBM patients with a potential positive effect on overall survival. Recent clinical trials showed potential therapeutic efficacy and minor side effects, thus opening a promising era for GBM medical care (27–30). Challenges remain, however, in developing catheter systems that will allow for effective coverage of the entire tumor volume while minimizing unwanted backflow or spillage into regions of normal brain tissue. In addition, further improvements are desirable in the chemical and biological properties of the molecular vectors carrying the alpha emitters, in terms of stability, specificity to cancer cells, and ability to diffuse freely inside the tumor.

In this work, we consider the combination of TMZ and BEV with another modality using alpha particles: diffusing alpha-emitters radiation therapy (DaRT). DaRT is a unique method that allows the treatment of solid tumors by alpha particles using implantable sources (“seeds”) carrying low activity levels of ^224^Ra (*t*_1/2_ = 3.63 days) a few nanometers below their surface (31, 32). Once inside the tumor, the DaRT seed continuously releases radon-220 (^220^Rn, *t*_1/2_ = 55.6s) atoms by recoil into the tumor tissue. The process continues as long as the seed remains inside the tumor, and its rate decays exponentially with the half-life of ^224^Ra, such that ~75% of the total dose is delivered within the first week, and ~95% in 16 days. ^220^Rn, a noble gas, diffuses freely as a free atom in the vicinity of the seed, decaying by alpha emission (*E_α_* = 6.29 MeV) up to ~2–3 mm away from its surface, followed by additional alpha emissions by ^216^Po (*t*_1/2_ = 0.145s, *E_α_* = 6.78 MeV) at the same location, and by the alpha-emitting daughters of ^212^Pb (*t*_1/2_ = 10.64*h*) − ^212^Bi (*t*_1/2_ = 60.55min, *Ē_α_* = 6.06 MeV) and ^212^Po (*t*_1/2_ = 0.30μs, *E_α_* = 8.78 MeV). The diffusion process creates a continuous “kill region” of a therapeutic alpha-particle dose ( ~ 10–20 Gy) with a typical diameter of ~3–5 mm, for a seed carrying a few microcurie of ^224^Ra (31, 33). This suggests positioning seeds at a spacing of a few millimeters from each other. The effective diameter of the high-dose region around the seed depends on the tumor type and is thought to be affected by the presence of active vasculature and necrotic domains (32). When treating the tumor with an array of seeds, the dose falls off to negligible levels ~2–3 mm away from the outermost seeds (34, 35). ^212^Pb atoms entering the bloodstream are trapped by red blood cells (31, 36) and may thus “leak” out from the tumor, with subsequent uptake in various organs. Estimates from a biokinetic model, based on the lead and bismuth models of the International Commission on Radiological Protection (ICRP), indicate that the maximal tolerable activity of ^224^Ra on the DaRT seeds, limited by the dose to the kidneys and red bone marrow, is a few millicurie (36).

Preclinical studies have demonstrated the capabilities of DaRT to produce *in vivo* anti-tumor responses in multiple tumor-bearing mice models (31, 33, 37–41) both directly, and by stimulating an immune response (42–45). This was successfully translated into a clinical trial of recurrent and locally advanced squamous cell carcinoma of the skin and head and neck, in which the DaRT treatment achieved a 100% tumor response (with all tumors shrinking by 30%–100%), with 78.6% complete response (macroscopic disappearance) of the tumors, and with minimal adverse effects (46). The calculated kidney and red bone marrow dose in all patients was on the cGy level, with no signs of systemic toxicity. Preclinical studies have also demonstrated the superiority of DaRT when combined with chemotherapy in squamous cell carcinoma (40), pancreatic (33), and colon cancer xenograft models (38). Yet, in GBM xenografts, the effect of DaRT was only investigated as a monotherapy (39) and the effective inhibition of tumor development was not conducted with the standard clinical care of GBM, an essential step for translating the preclinical results into clinical therapy.

In the context of treating recurrent GBM, DaRT has the potential advantage of allowing for controlled geometrical coverage of the tumor volume, by the use of dedicated applicators that enable the insertion of multiple seeds from a single entry point. With each seed creating a well-defined, high-dose region in its few-millimeter vicinity, this approach can permit quantitative treatment planning, as well as real-time identification of potential cold spots and their mitigation. Unlike intratumoral injection [of either TAT vectors, or vehicles carrying beta emitters such as ^186^Re (47) or ^90^Y (48)], which is subject to uncertainty resulting from uncontrolled pressure gradients and backflow, the single-atom diffusion process in DaRT (in particular, that of ^220^Rn) is more predictable and less prone to inadvertent local toxicity. While the diffusing atoms do not bind specifically to cancer cells as in TAT, their continuous distribution in the seed vicinity and the rapid fall-off of the dose outside of the treatment region can be regarded as “geometric targeting”. Compared to interstitial treatments for using ^125^I (49) or ^192^Ir (50), which also provide controlled geometric coverage of the target volume, the use of alpha particles in DaRT is immune to hypoxia, and the rapid fall-off of the dose is expected to lead to reduced toxicity to normal brain tissue. Lastly, the production of the ^224^Ra-loaded DaRT seeds relies on thorium-228 (^228^Th), which is readily available at large quantities and has a half-life of 1.9 years, allowing for a steady, cost-effective, and large-scale manufacturing of seeds.

In the current study, we investigated the potential of DaRT in combination with BEV or TMZ to eradicate GBM xenografts. In addition, the cytotoxicity of alpha radiation in combination with TMZ and the effect of alpha radiation on VEGF secretion by U87 cells were studied *in vitro*. Finally, we also studied the effect of BEV on the diameter of the high-dose region surrounding the DaRT seed and on the rate of ^212^Pb clearance from the tumor. The results of this study are a first step in investigating the potential use of DaRT in combination with TMZ and/or BEV in the treatment of recurrent GBM.

## Materials and methods

### Animals

All animal experiments were carried out in accordance with the government and institution guidelines and regulations (Ethics approval 01-20-055, IL-80-10-2020E) and with the National Institute of Health *Guide for the Care and Use of Laboratory Animals* (NIH Publications No. 8023, revised 1978). Athymic nude mice, strain Hsd: Athymic-Nude-Foxn1-nu female mice (24–30 g, 12–15 weeks) were obtained from Envigo (Jerusalem, Israel) and kept in the animal facility of Tel Aviv University or Ben-Gurion University.

### Tumor cell lines

U87 human GBM cell line (ATCC) cells were grown in MEM-NEAA containing L-glutamine (Cat. # 01-040-1A, Biological Industries, Kibbutz Beit Haemek, Israel), supplemented with 10% non-heating inactivated fetal calf serum, penicillin (100 U/ml), and streptomycin (100 μg/ml) (Biological Industries, Kibbutz Beit Haemek, Israel), in a humid incubator at a temperature of 37°C and 5% CO_2_.

### Tumor cell inoculation

U87 cells were inoculated intracutaneously to the right lower lateral side of the back in a concentration of 5 × 10^6^ cells/100 μl of HBSS (Biological Industries, Kibbutz Beit Haemek, Israel), using a 29-gauge needle. The procedure was performed under anesthesia (xylazine 10 mg/kg, ketamine 100 mg/kg, i.p.(or isoflurane (5% isoflurane for initial anesthesia and 3% isoflurane for the DaRT insertion procedure).

### DaRT seeds preparation and insertion

DaRT seeds were made of 6.5-mm-long stainless steel (316 LVM) tubes with an outer diameter of 0.7 mm and an inner diameter of 0.4 mm, loaded with **^224^** Ra atoms, following an electrostatic collection process similar to that described in (31). To prevent radium detachment from the surface, the seeds were subsequently coated by a 1-μm-thick biocompatible polymer layer made from polydimethylsiloxane (PDMS-silicone adhesive, MED2-4213), which allows ^220^Rn atoms to be released into the tumor tissue. The ^220^Rn desorption probability (the probability that a ^220^Rn atom is emitted from the seed following a decay of ^224^Ra) was 40%–45%, unless mentioned otherwise. The ^224^Ra activity was 2 or 3 μCi (~75 or 110 kBq). Seeds, either loaded with **^224^** Ra or inert, were placed near the tip of a 19- or 18-gauge needle connected to a seed insertion applicator (essentially a modified syringe, see **Figure 2**). A single radioactive or inert seed was inserted into the tumor under anesthesia (xylazine 10 mg/kg, ketamine 100 mg/kg).

### Drug preparation and storage

TMZ (cat. # S1237, Selleckchem, TX, USA) was dissolved in DMSO and prepared in aliquots of 20 mg/ml DMSO. Stock solution was stored at −80°C. A solution of BEV at 4 mg/ml (Avastin^®^ Genentech, CA, USA) was stored at 2–8°C in a refrigerator in the dark. Control antibody human IgG1 (Sigma, MO, USA, cat. # I4506) was stored prior to reconstitution at 2–8°C. After reconstitution, aliquots were stored at –20°C.

### *In vivo* drug treatments

For the *in vivo* studies, TMZ was dissolved in 5% DMSO and 30% PEG300 in ddH_2_O (51), and 200 µl of 1 mg/kg TMZ was injected i.p. into the mice; 5% DMSO and 30% PEG300 in ddH_2_O was used as a vehicle control. Treatment started 1 day after DaRT insertion (day 0) for a total of nine doses (days 1–4 and 6–10). BEV (5 mg/kg) was administrated i.p. at 90–130 µl/mouse, starting from day 5 after DaRT insertion (on days 5, 7, 9, 12, 14, 16, 19, 21, and 23, where day 0 is the day of DaRT seed insertion) unless mentioned otherwise in the *Results* section.

### *In vivo* tumor measurements

Local tumor growth was determined two to three times per week after seed insertion by measuring three mutually orthogonal tumor diameters (*D*_1_, *D*_2_, *D*_3_) using a digital caliper, along with radioactivity measurement using a Geiger counter to confirm the presence of a DaRT seed in the tumor. Tumor volume was estimated as $\frac{\pi }{6}D_{1}D_{2}D_{3}$ . Animal welfare was monitored (body weight measurements and animal behavior) and survival was recorded on the day of tumor measurements. Animals were sacrificed and considered dead on the day the tumor size reached the ethical limit (1,500 mm^3^ for BEV experiments or 15 mm for TMZ experiment). The experiments were terminated when in all groups at least one animal died, unless mentioned otherwise.

### *In vitro* irradiation setups

**Figure 1:**
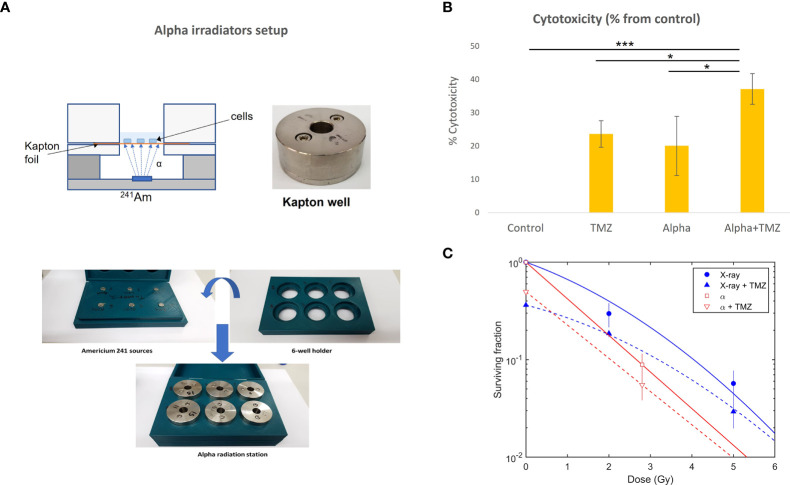
Cytotoxic effect of alpha and x-ray radiation, TMZ, or the combined treatment on U87 cells. **(A)** Alpha irradiation setup. Cells were seeded on a Kapton foil and exposed to alpha particles emitted by a ^241^Am source placed, in air, 9.6 mm below. **(B)** The effect of 1.4 Gy of alpha particles, 300 µM TMZ (72 h), or the combination of the two. Cytotoxicity is calculated as the difference between viability following treatment (% from control) and that of the control (100%) and is expressed as mean ± standard deviation of three independent experiments. *p_t-test_<0.05, ***p_t-test<_0.0005. **(C)** Colony formation assay, with cells irradiated by alpha particles or x-rays with and without TMZ (15 µM of TMZ for 24 h prior to irradiation).

U87 cells were exposed *in vitro* to alpha particles with and without TMZ in a viability assay, and to both alpha particles and x-rays, with and without TMZ, in a colony formation assay. *In vitro* exposure to alpha particles was done in irradiation stations (“Kapton wells”, **Figure 1A**), each equipped with a 3.8-μCi (140 kBq) ^241^Am source, which, due to partial screening, had an alpha emission rate of 50 ± 2.5 kHz. Cells were seeded on a 7.5-μm thick Kapton foil (polyamide film PRN-IF70, Pornat, Israel) assembled between two cylindrical stainless steel parts with an exposed area 9 mm in diameter, as previously described (52). The cells, covered by a medium, were positioned 9.6 mm above the ^241^Am source in air and exposed to a controlled flux of alpha particles, at an average dose rate of 0.10 Gy/min. The dose rate was estimated by measuring the spectrum and emission rate of alpha particles from the ^241^Am source with an alpha-particle spectrometry system, supplemented by a Monte Carlo simulation of the full setup, using a dedicated MATLAB script utilizing SRIM 2013 (53). The calculated mean alpha-particle energy, when passing through the cell layer, was 2.7 MeV, with an LET of 130 ± 3 keV/μm (where the variations reflect the radial position across the foil). For x-ray exposure, cells were seeded in six-well plates and irradiated with a Faxitron cabinet x-ray system MultiRad 160 (Faxitron X-ray Corporation, IL, USA).

### *In vitro* treatment for viability assay

Cells were seeded in the Kapton wells in five replicates for each treatment. Twenty-four hours post seeding, the medium was replaced by a fresh complete medium or by a complete medium containing 300 µM TMZ dissolved in DMSO, or DMSO as control (54). The TMZ concentration and alpha dose were pre-calibrated and chosen to achieve 80% viability following monotherapies. Exposure to alpha particles at a single level (1.4 Gy) was performed 2 h after the medium was replaced. The cells then remained in incubation with or without TMZ for an additional 70 h and were then subjected to a PrestoBlue viability assay. The experiment was repeated three times.

### PrestoBlue viability assay

To evaluate cell viability, PrestoBlue reagent (A13261, Invitrogen, CA, USA) was used as follows: The cell medium was removed and a 240-µl phenol-red-free DMEM medium (Biological Industries, Kibbutz Beit Haemek, Israel) containing 10% PrestoBlue was added to each Kapton well. After 30 min of incubation, duplicates of 100 µl of medium containing PrestoBlue from each well were transferred to a 96-well plate. Absorbance was measured either using an EMax Plus microplate reader (Molecular Devices, CA, USA) at 570 and 595 nm, or a Multiskan Spectrum spectrophotometer (Thermo Scientific, MA, USA) at 570 and 600 nm (the latter was used in the VEGF experiment, see below). Viability values represented the following calculation (according to the manufacturer’s instructions): (OD_570_-blank) − (OD_600_-blank), where OD stands for optical density.

### Colony formation assay

In the colony formation assay, x-ray irradiation was done at 2, 5 and 10 Gy. Alpha-particle irradiation was done at 2.8, 7, and 14 Gy. For alpha particles, in practice, only the 2.8-Gy level was useful, as survival at higher doses was dominated by cells evading direct hits, artificially leading to unrealistic survival estimates. For alpha-particle irradiation, 12,000 cells were seeded in four to six Kapton wells for each irradiation level and covered by 300 µl of medium. For x-ray irradiation, 1.8 × 10^5^ cells were seeded in two wells of a six-well plate and covered by 2 ml of medium. One day after seeding, the medium was changed to a fresh complete medium containing either 15 µM TMZ or DMSO (control). Twenty-four hours later, the medium was again replaced by fresh complete medium to stop the TMZ treatment. Cells were immediately exposed to alpha or x-ray radiation. Shortly after irradiation, cells were dissociated, pooled, and seeded in different concentrations in duplicates in six-well plates for an incubation period of 10–15 days to allow the formation of colonies. Colonies were then fixed in methanol and stained with crystal violet. Colonies with more than 50 cells were counted as viable. Plating efficiency and survival fraction were calculated based on three independent experiments for alpha particles and for x-rays. Survival data for x-ray irradiation were fitted by *S(D)* = exp(-*αD*-*βD* ^2^), and for alpha irradiation by *S(D)* = exp (-*D*/*D*_0_) (with an additional constant term for the combination with TMZ) using MATLAB (MathWorks, ver. R2021B).

### VEGF ELISA assay

To determine VEGF levels in cell culture-conditioned medium, medium was collected from culture wells and frozen 72 h after treatment start. VEGF secretion was determined using quantitative-sandwich ELISA kit (Human VEGF DuoSet ELISA, R&D Systems, #DY293B) following manufacturer’s instructions. Normalization of VEGF secretion was performed proportionate to the number of cells by dividing the result of VEGF secretion by % viability (from control) according to a PrestoBlue viability assay.

### Autoradiography of DaRT-treated tumors and ^212^Pb leakage probability measurements

A single DaRT seed (6.5 mm length, 0.7 mm outer diameter), carrying 3 μCi ^224^Ra, was inserted to the center of a mice-borne U87 tumor 7–20 days after tumor inoculation, when the tumor transverse diameter was ~6–0 mm. Four to five days later, the tumor was excised (as a whole) and cut in two halves, at the estimated location of the seed center, perpendicular to the seed axis. The seed was then pulled out using surgical tweezers and placed in a water-filled tube for subsequent measurement by a well-type NaI(Tl) detector (Hidex Automatic Gamma Counter). The tumor was kept for 1 h at −80°C. It was then taken, in dry ice, for measurement in the same gamma counter to determine the ^212^Pb activity it contains, by focusing on the ^212^Pb 239 keV gamma line. The measurements of the seed and tumor activity were used to determine the ^212^Pb leakage probability from the tumor (i.e., the probability that a ^212^Pb atom released from the seeds leaks out from the tumor through the blood before its decay) following the procedure described in (31, 32).

Immediately after the gamma measurement, both halves of the tumor were subjected to histological sectioning using a LEICA CM 1520 cryostat (Buffalo Grove, IL, USA). Sections were cut at 250–300 μm intervals with a thickness of 10 μm, and were then placed on positively charged glass slides, fixed with 4% paraformaldehyde (sc-281692, Santa Cruz Biotechnology Inc., Dallas, Texas, USA) and rinsed twice with PBS. Typically, there were 5–15 sections per tumor, spanning a length of 1.5–5 mm. Shortly after their preparation, the glass slides were placed, faced down, for a duration of 1 h, on a phosphor imaging plate (Fujifilm TR2040S) protected by a 12-μm Mylar foil and enclosed in a light-tight casing. Alpha particles emitted from the sections in the decays of ^212^Pb progeny atoms, ^212^Bi and ^212^Po, penetrate through the foil and deposit energy in the active layer of the phosphor imaging plate. Immediately after exposure, the plate was read out by a phosphor-imaging scanner (Fujifilm FLA-9000).

For each tumor section, the result was a two-dimensional intensity map, proportional to the local ^212^Pb activity. After performing a deconvolution process with the known point-spread-function of the system to deblur the recorded image (31), the intensity (in units of photo-stimulated luminescence) was converted to ^212^Pb activity using suitable ^212^Pb calibration samples. The local ^212^Pb activity was then used to provide a gross estimate of the size of the therapeutically affected region—the *effective diameter*—based on the approximate procedure described in (31). Briefly, the recorded local ^212^Pb activity serves to calculate the asymptotic macroscopic dose that would have been delivered by the alpha emissions of ^212^Bi and ^212^Po (in local secular equilibrium with ^212^Pb) if the seed were to be left inside the tumor indefinitely (in practice, for >3 weeks), assuming a uniform time dependence of the activity throughout the tumor volume. By calculating the total area corresponding, in a given tumor section, to an asymptotic ^212^Bi/^212^Po alpha dose larger than 10 Gy, A(*D*_B_ *_i_*_P_ *_o_* > 10 Gy), the effective diameter is defined by: *d_eff_* = 2[A(D_B_ *_i_*_P_ *_o_* > 10 Gy)/π]^1/2^. We stress that this parameter should be strictly considered as a gross indicator for the spatial spread of the alpha-emitting daughters of ^224^Ra inside the tumor. The 10-Gy dose is chosen as a convenient reference for actual therapeutic alpha-particle doses that are expected to be in the range ~10–20 Gy.

The same histological sections measured on the imaging plate were later stained with hematoxylin–eosin (H&E) (G-biosciences, St Louis MO, USA) for tissue damage detection. H&E staining was correlated with the activity distribution measurements. The pictures were taken using a Panoramic scanner (3D HISTECH Ltd., Budapest, Hungary).

### Immunohistochemical staining and analysis

To determine the presence and expression of CD31, slides were dried and incubated in cold acetone for 20 min and then washed in PBS. Staining was performed with a Leica Bond-III Automated Stainer according to manufacturer’s instructions using the primary antibody for CD31 (ab281583, Abcam, dilution 1:200). The immunostaining area of CD31 was measured using ImageJ. The red channel was separated from blue and converted to grayscale, and the background was subtracted. The values of the monochromatic images were then measured and expressed as percentage of area. To quantify the CD31 staining regions, three areas from each section (ROIs) were selected from non-necrotic areas, and their average was calculated per each slide. One tumor was excluded from the analysis according to Grubb’s test.

### Statistical analysis

Tumor volume over time was assessed and compared between the groups using repeated measures analysis of variance with false discovery rate (FDR) correction for multiple comparisons. The cubic root transformed volume was modeled as a function of group, day (categorical), and the day × group interaction with baseline volume entered as a covariate. The model estimated means (least squares means) and confidence intervals were estimated from the interaction term for each day per group and were back transformed to the volume. Each experiment was analyzed until the time point at which the first animal died. The days for which the differences were significant were mentioned in the *Results* section and the *p*-value range was presented. Tumor volume graphs included data until the first animal in the last group died. Time to 5-fold change in tumor volume data are depicted by a Kaplan Meier plot; two curves are compared with a Log-rank test with *p*-values adjusted for multiple comparisons using the FDR method. For effective diameter and leakage analysis, ANCOVA was performed. Pearson’s correlation coefficient was used to assess correlation between effective diameter and leakage. Grubbs’ test was used to identify outliers. A *p*-value< 0.05 was considered statistically significant. The above-mentioned analyses were performed using SAS V9.4 (SAS Institute, Cary NC, USA). For the difference between the means of two treatments in *in vitro* and *ex vivo* (CD31 IHC and effective diameter) studies, a two-tailed Student’s *t*-test was used (Excel, Microsoft).

## Results

### Cytotoxicity of TMZ in combination with alpha radiation is higher than the cytotoxicity of each treatment alone

As discussed above, the combination of alpha-particle irradiation with TMZ was studied both in a viability assay and in a colony formation assay, where the latter also included an arm replacing alpha particles by x-rays. In the viability assay, cells were either treated with 300 µM TMZ (for 72 h), exposed to a dose of 1.4 Gy of alpha-particle flux, or both. Viability scores were calculated for each treatment relative to the untreated control group (% from control). Cytotoxicity was expressed as the difference between viability following treatment and that of the control. The results are shown in **Figure 1B**. Both TMZ and alpha radiation exerted a cytotoxic effect on U87 cells compared to untreated cells (*p*< 0.001,<0.05, or <0.0005 for TMZ, alpha irradiation, or the combination vs. control, respectively). No significant difference was observed between alpha irradiation and TMZ treatments. The combinational treatment nearly doubled the cytotoxicity relative to each of the treatments alone (*p*< 0.05, for each monotherapy vs. combination).

In the colony formation assay, cells were exposed to 0, 2.8, 7, and 14 Gy of alpha particles and x-rays, following 24 h of TMZ. The results are shown in **Figure 1C**. As noted above, for the alpha-particle case, only the control and 2.8 Gy points were used in the analysis, as colony formation after exposure to 7 and 14 Gy was unrealistically high (~4% and ~3%, respectively, compared to an expected respective survival of ⪅1% and ⪅0.01%). We suspect that this resulted from cells entering a “sheltered” region in a peripheral gap between the Kapton foil and stainless steel cover.

Without TMZ, the mean lethal dose for alpha particles was estimated as *D*_0_= 1.2 ± 0.2 Gy. Compared to x-rays, this translated to *RBE*_10_ = 1.5 ± 0.3 (i.e., at 10% survival). The addition of TMZ reduced the surviving fraction by ~40%–50% for both x-ray and alpha-particle irradiation.

### DaRT combined with TMZ delayed tumor development more than the monotherapies

**Figure 2:**
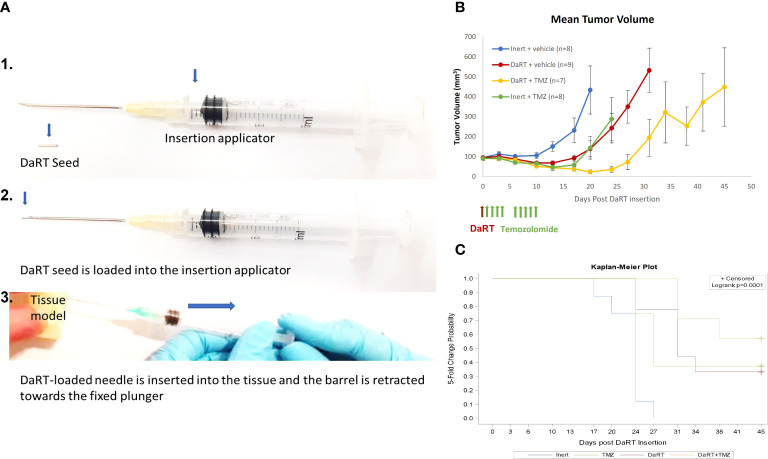
Tumor growth following treatment with DaRT, TMZ, or the combined treatment. U87-bearing mice (~90 mm^3^) were treated with a 75-kBq DaRT seed or inert seed on day 0, followed by nine doses of 1 mg/kg TMZ i.p. on days 1–4 and 6–10. **(A)** DaRT seed and insertion applicator. **(B)** Mean tumor volume ± SEM. The combined treatment significantly reduced tumor growth compared to control, DaRT, or TMZ monotherapies (*p <* 0.0005, *p <* 0.0005, *p <* 0.05). **(C)**. The time to reach fivefold the starting tumor volume was prolonged for animals receiving DaRT, TMZ, or the combined treatment compared to the control group (*p <* 0.005, *p <* 0.05, *p <* 0.001, respectively).

Given the observed cytotoxic effect of DaRT combined with TMZ in cultured U87 cells, the potential of such a combination was also tested *in vivo*. The number of DaRT seeds and TMZ dose were chosen so that no complete elimination of the tumor (46) will occur in the majority of animals following monotherapies. U87 tumor cells were inoculated, and tumors were allowed to reach an average size of 7 mm (longest diameter) for 7 days. On day 7, a single DaRT or an inert seed (non-radioactive control) was inserted into each tumor (**Figure 2A**). One day post seed insertion, mice were administrated with TMZ or vehicle (control) for a total of 9 doses. One animal from the combinational group was excluded due to seed loss between days 3 and 6. Tumor growth among DaRT (*n* = 9), TMZ (*n* = 8), or the combined treatment (*n* = 7) groups was reduced when compared to the control (*n* = 8) group (day 20, *p<* 0.005; days 13–20, *p<* 0.001–0.0001; days 17–20, *p<* 0.0005–0.0001, respectively). The combined treatment group showed the highest tumor development retardation effect. No significant difference was seen in tumor growth between the DaRT and TMZ monotherapy groups at any time point. Tumor growth retardation induced by DaRT or TMZ was significantly different from that of the combined treatment (day 20, *p<* 0.0005 or *p<* 0.05, respectively) (**Figure 2B**). The time to reach fivefold of the starting tumor volume was prolonged for animals treated with DaRT, TMZ, or the combined treatment compared to the control group (*p<* 0.005, *p<* 0.05, *p<* 0.001 respectively) (**Figure 2C**).

### Alpha radiation does not affect the secretion of VEGF from U87 cells

As mentioned in the *Introduction*, low-LET radiation, such as x-ray (14), can upregulate VEGF secretion. This may result in a reduced efficiency of BEV treatment when given in combination with radiation therapy. Here, we tested whether alpha irradiation leads to the same effect, by exposing U87 cells to alpha particles (up to 2.8 Gy) and measuring their VEGF secretion by an ELISA assay. Due to the cytotoxicity of the treatment, cell viability, scored by the PrestoBlue viability assay after medium removal, was used to normalize VEGF secretion proportionally to the number of live cells. No significant changes in VEGF secretion by U87 cells was observed (*p*-value = 0.9, 0.7, and 0.7 for control vs. 0.7 Gy, 1.4 Gy, and 2.8 Gy, respectively) (**Table 1**).

**Table 1:** VEGF secretion by U87 cells following alpha irradiation (normalized to % viability).

Dose (Gy)	VEGF level (pg/ml)	Standard error	*p*-value (vs. control)
**0 (control)**	1,899.6	421.1	
**0.7**	1,844.3	517.4	0.9
**1.4**	1,692.1	377.5	0.7
**2.8**	1,703.0	304.6	0.7

U87 cells were treated with 0.7, 1.4, and 2.8 Gy alpha irradiation and VEGF secretion was determined using the ELISA assay 72 h post-treatment. Secretion was normalized for cell viability. VEGF concentration is expressed as the mean of three independent experiments. No significant enhancement of VEGF secretion was observed.

### DaRT insertion prior to BEV administration attenuates tumor growth of GBM xenografts compared to either monotherapy

**Figure 3:**
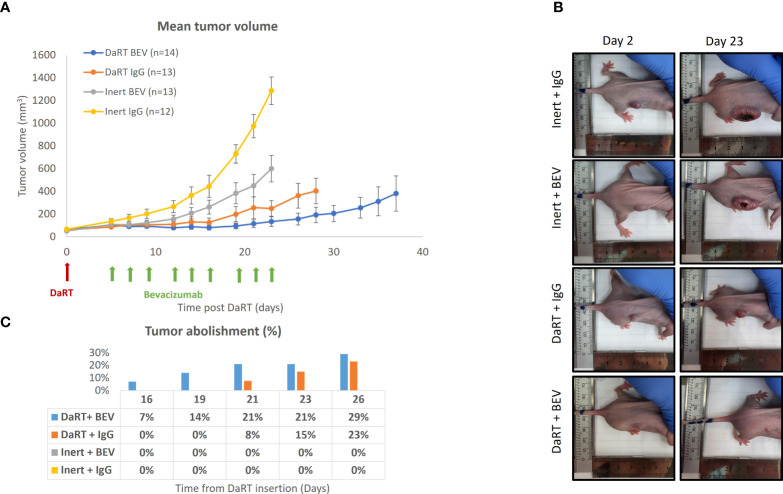
Tumor growth following the combination treatment of DaRT and BEV. U87-bearing mice (~90 mm^3^) were treated with a 110-kBq DaRT seed or inert seed on day 0, followed by nine i.p. injections of either BEV or IgG control (5 mg/kg) on days 5, 7, 9, 12, 14, 16, 19, 21, and 23 (three times per week for three consecutive weeks). Results represent cumulative data of two independent experiments. **(A)** Tumor volume ± SEM: *p<* 0.05, *p <* 0.05, *p <* 0.001 for the combined therapy vs. BEV, DaRT, or control, respectively. **(B)** Representative photographs of one animal from each treatment group on day 2 and day 23 post DaRT treatment. **(C)** Percent of tumors that were eradicated in each treatment group. These mice were followed up for a period of over 3 months, with no recurrence.

Given the observation that exposure to alpha particles does not lead to an increase of VEGF secretion in U87 cells, we hypothesized that the antiangiogenic function of BEV will not decrease in combination with alpha radiation, allowing additivity by preventing the potential renewal of GBM tissue following DaRT-induced ablation. To test the effect of DaRT in combination with systemic BEV *in vivo*, two treatment regimens were used. In the first, U87 xenografts were transplanted and allowed to grow for 9 days to an average size of ~6.5 mm (longest diameter). Thereafter, each tumor was treated with either a single DaRT or inert seed. BEV or IgG control treatment began 5 days post seed insertion. DaRT (*n* = 13) or BEV (*n* = 13) as a standalone treatment provided significant attenuation in tumor growth compared to the control (*n* = 12) group (days 12–23, *p<* 0.01–0.0001; days 19–23, *p<* 0.01–0.0001, respectively). DaRT-treated tumors were significantly smaller compared to the BEV-treated group (days 14–23, *p<* 0.05–0.0001). A significant effect on tumor growth was observed in the combined therapy group (*n* = 14) compared to BEV (days 12–23 *p<* 0.05–0.0001), DaRT alone (days 19–23, *p<* 0.05), or control (days 12–23, *p<* 0.001–0.0001) (**Figures 3A, B**). Twenty-six days after DaRT insertion, 29% of the tumors in the DaRT+BEV group were eradicated (4 animals out of a total of 14) and 23% of the tumors in the DaRT-alone group were eradicated (3 animals out of a total of 13). Tumors that underwent a complete response were declared as cured as they did not recur for a period of over 3 months from the day of the DaRT treatment (**Figure 3C**).

### Early administration of BEV relative to DaRT led to a higher effect of the combination relative to either monotherapy in larger tumors

**Figure 4:**
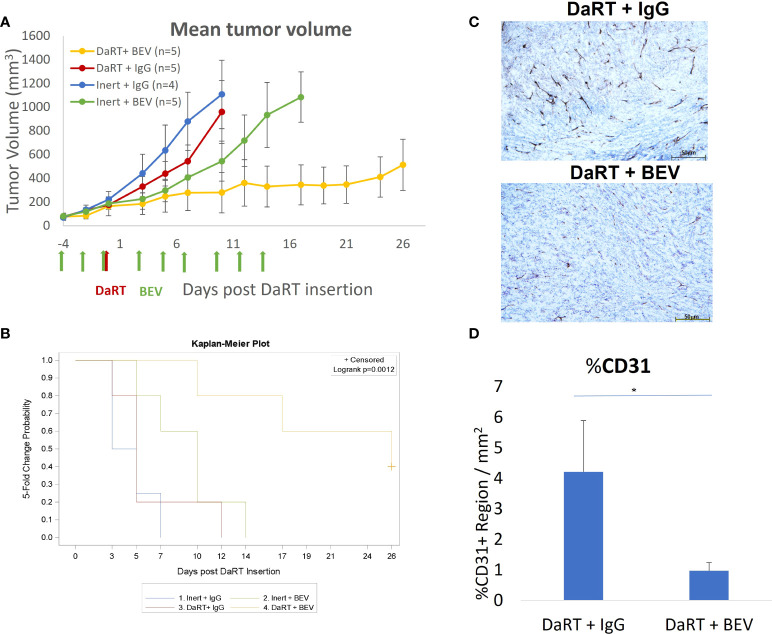
Tumor growth following early administration of BEV relative to DaRT. U87-bearing mice were treated with either BEV or IgG control (5 mg/kg) on days −4 to 14. Four days after the first BEV dose, each tumor was treated with either a single 110-kBq DaRT seed or an inert seed. **(A)** Tumor volume vs. time (*p<* 0.005 for the combinational vs. control or vs. each of the monotherapies). **(B)** Time to reach fivefold of the initial tumor volume (*p <* 0.05, combination vs. control). **(C)** Representative images of CD31 immunohistochemical staining of DaRT-treated U87 tumors following IgG or BEV (*n* = 4/group) treatments. Scale bars represent 50 μm. **(D)** Quantification of area percentage of CD31 staining using ImageJ, showing the mean ± SD of the averaged % of CD31+ region per three representative areas (ROI) in a single specimen. *p < 0.01 DaRT+IgG vs. DaRT+BEV.

The results shown in **Figure 3** indicate that in 6- to 7-mm tumors, the DaRT treatment was the dominant factor affecting tumor retardation and elimination, and the combination with BEV significantly increased tumor control. To further support a potential synergy between DaRT and BEV, we used a different treatment protocol in which a single DaRT seed was inserted into larger tumors relative to the seed size (~9 mm average in their longest diameter). Such a setting prevents the alpha-emitting atoms from covering the entirety of the treated tumors with therapeutic levels of radiation dose. In this protocol, BEV administration (same treatment parameters as in **Figure 3**) preceded DaRT seed insertion by 4 days. In addition, the changes in blood vessel structure by BEV in this treatment protocol were validated four days after DaRT implantation (activity = 110 kBq, ^220^Rn desorption probability ~25%) using CD31 staining. U87 xenografts were transplanted and allowed to grow to an average size of 6.5 mm (longest diameter) when the BEV treatment began (see **Figure 4**). Four days after the first BEV dose, when the average tumor length (longest diameter) reached ~9 mm, each tumor was treated with either a single DaRT or an inert seed. Tumor development follow-up ended on day 26 as some of the tumors were unmeasurable due to pronounced necrosis. Monitoring tumor growth revealed that DaRT (*n* = 5), BEV (*n* = 5), or the combination treatment (*n* = 5) delayed tumor growth compared to control (*n* = 4) (day 7, *p<* 0.05; days 3–10, *p<* 0.01–0.0005; days 3–10, *p<* 0.005–0.0001). BEV reduced tumor growth compared to DaRT (day 10, *p<* 0.01). The combination treatment showed significant growth delay compared to the DaRT or BEV (days 3–10, *p<* 0.05–0.0001; day 10, *p<* 0.005; respectively) alone (**Figure 4A**). The time to reach fivefold the initial tumor volume was increased in the combined treatment vs. DaRT, BEV, or control (*p<* 0.05) (**Figure 4B**). A significant reduction in CD31 staining (*p<* 0.01) validated the effect of BEV on the DaRT-treated blood vessels (**Figures 4C, D**). Notably, one out of the five animals treated with DaRT+BEV experienced a complete eradication of the tumor. This phenomenon was not recorded in other treatment groups.

### BEV increases the spread of alpha emitters in the tumor by reducing the clearance of ^212^Pb through the blood

**Figure 5:**
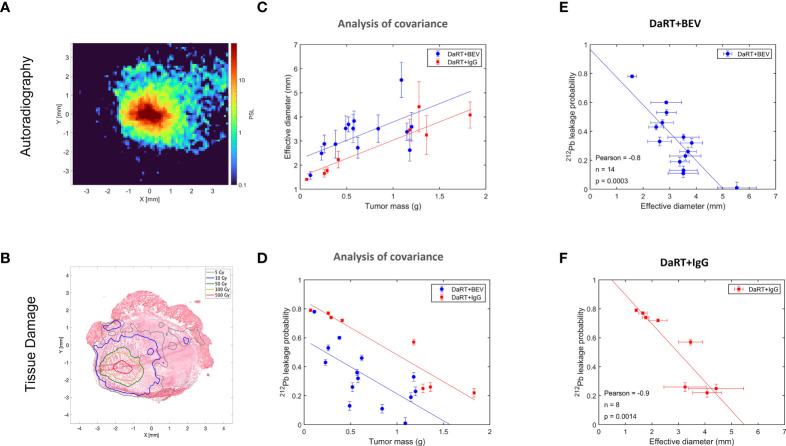
Effect of BEV on the intra-tumoral spread of 212Pb and its clearance from the tumor through the blood. **(A)** A typical autoradiography image of a U87 tumor treated by a single DaRT seed, in raw photo-stimulated luminescence (PSL) units. **(B)** Same tumor section, with the PSL map translated to estimated dose, overlaid on an image of the H&E-stained section. **(C)** Effective diameter of the region subject to an estimated macroscopic ^212^Bi/^212^Po alpha dose of >10 Gy as a function of the tumor mass, for tumors treated by DaRT+BEV and by DaRT+IgG as control. **(D)** The ^212^Pb leakage probability as a function of the tumor mass for the DaRT+BEV/IgG groups. **(E, F)** Correlation between the effective diameter and ^212^Pb leakage probability for the two groups.

As discussed in (32), the physics model of DaRT predicts that the spread of ^212^Pb inside the tumor decreases with increasing rate of its clearance through the blood. Here, we quantified the spread by the effective diameter of the region in which the local measured ^212^Pb activity translated to an estimated macroscopic alpha dose of >10 Gy by the alpha emissions of ^212^Bi and ^212^Po (note that this does not include the contribution of the two alpha particles emitted by ^220^Rn and ^216^Po). The clearance rate of ^212^Pb is quantified by its leakage probability from the tumor. Since BEV affects the tumor vasculature, its combination with DaRT could modify the leakage probability of ^212^Pb, and therefore also its spatial spread inside the tumor. To investigate this, autoradiography experiments and measurements of the ^212^Pb leakage probability were performed on U87 tumors over a mass range ~0.1–2 g, where the tumors were treated with a single DaRT seed combined with either BEV or IgG as control, following the same treatment regimen employed in the efficacy experiments described in **Figure 4**. **Figure 5A** shows an example of an autoradiography image of a treated tumor, and **Figure 5B** demonstrates an overlay of the estimated ^212^Bi/^212^Po dose on the H&E-stained image of the same section. **Figure 5C** shows the dependence of the effective diameter on the tumor mass for the two groups. While in both groups the effective diameter increases with the tumor mass, the effective diameter in the case of DaRT+BEV is, on average, larger by ~0.7 mm compared to DaRT+IgG for the same tumor mass. A covariance analysis showed a statistically significant difference (*p<* 0.05) between the two groups. **Figure 5D** shows the dependence of the ^212^Pb leakage probability on the tumor mass for the two groups. In the DaRT+BEV group, the leakage probability is on average smaller by ~25% (in absolute value) compared to the DaRT+IgG group, with a statistical significance of *p<* 0.0005. The effective diameter was negatively correlated with the leakage from the tumor in both groups (**Figures 5E, F**, *p<* 0.005).

## Discussion

The current study demonstrated a superior effect of DaRT in combination with standard-of-care drugs (TMZ and BEV) in mice-bearing GBM xenografts, relative to the effect of the single-treatment modalities. As a first step, we examined the direct effect of the standard chemotherapy, TMZ, in combination with alpha-particle irradiation on U87 cells. One could expect that the combination treatment would yield a lower cytotoxic effect than the sum of the monotherapies, due to a subpopulation of cells affected by both TMZ and alpha particles. Nevertheless, alpha irradiation in combination with TMZ was actually shown to double the cytotoxic effect of each of the monotherapies on U87 cells in culture. This may suggest that for cells that would have otherwise received a sublethal dose of either TMZ or alpha irradiation and survived these monotherapies, the DNA breaks caused by the combination of TMZ (55, 56), together with the complex DNA damage produced by alpha particles (57), lead to unrecoverable damage to the DNA. In addition, this combination was advantageous compared to the combination of TMZ and x-rays as demonstrated by the lower surviving fraction in a colony formation assay. Finally, the enhanced tumor control of the combination treatment in GBM xenografts supported these observations and showed a superiority of combining DaRT with TMZ *in vivo*.

The previously reported ability of x-ray irradiation to upregulate VEGF expression and secretion in GBM cells (12–14) was not observed following alpha-particle irradiation. This may support using DaRT in combination with VEGF inhibition and may indicate that the DNA damage induced by alpha particles is unlinked to HIF-1-induced VEGF transcription (58, 59).

In the current study, two treatment regimens were used to evaluate the combination of DaRT and BEV *in vivo.* It was shown that when a single DaRT seed was used in a standard experimental protocol in relatively small tumors, in which the seed length was similar to the tumor’s longest diameter, the DaRT treatment was the main factor retarding tumor growth, whereas in relatively large tumors, where it would have been expected that a single DaRT seed would be less effective, the introduction of the BEV treatment prior to DaRT insertion yielded a pronounced effect of the combination treatment compared to both monotherapies, and a clear interaction between the treatments was demonstrated.

The underlying mechanism for this apparent synergy is not yet understood, and may, in fact, combine several complementary effects. First, the observation that BEV enhances the spatial spread of ^224^Ra progeny atoms, expressed by the effective diameter, inside the tumor and reduces the leakage of ^212^Pb into the bloodstream, may indicate a simple physical explanation, where more tumor cells are exposed to a lethal dose of alpha particles. A second possibility is that adding BEV to DaRT may reduce tumor regrowth by decreasing the vessel density and blood supply to surviving tumor cells and to potential glioma stem cell niches (60), thereby diminishing their re-establishment after the DaRT treatment. BEV administration may have also led to reduced interstitial pressure by reducing vessel leakiness (61). This could have assisted tumor shrinkage, bringing peripheral tumor cells closer to the seed. In addition, interstitial pressure within tumors is known to compromise drug extravasation across the capillary walls (62). It is yet unknown what is the effect of DaRT on the tumor microenvironment and whether it affects the availability of drugs such as TMZ or BEV to the tumor tissue. These questions should be further investigated in subsequent studies.

Traditional brachytherapy in the brain may lead to adverse effects such as intracranial arterial occlusion (63), hemorrhage, and radiation necrosis (64). Recently, ^131^Cs (half-life of 9.7 days) seeds, emitting ~30- keV x-rays, have received FDA clearance for brain tumors (65). It was suggested that this isotope would lead to less radionecrosis relative to ^125^I-based brachytherapy (66), whose 59-day half-life is considered a risk factor for late-stage adverse effects (66). The safety profile of DaRT in the brain is yet to be demonstrated. Nevertheless, several advantages of using DaRT may be expected: (1) the dose field is characterized by a rapid fall-off, dropping to negligible levels ~2–3 mm away from the outermost seeds, offering improved sparing of adjacent healthy brain tissue; (2) the cell-killing ability of alpha particles is largely unaffected by hypoxia, unlike low-LET radiation; (3) the half-life of ^244^Ra is relatively short (3.6 days), and thus, following radiation delivery, fewer late complications are anticipated; (4) DaRT does not counteract standard-of-care treatments, for example, by increasing VEGF secretion that may reduce the efficiency of BEV and enhance recurrence after treatment; furthermore, the present study even provides evidence that DaRT potentially synergizes with standard-of-care drugs, in particular with BEV. Thus, adding DaRT to such systemic therapies could potentially further delay the disease progression and extend the patient lifespan.

The presence of highly infiltrative cancer stem cells, in the peritumoral region of GBM, appears to play a key role in tumor growing and recurrence (67). High-LET radiation was previously shown to overcome radioresistance of glioma stem-like cells to low-LET radiation (68). Thus, it may be suggested that using DaRT for the treatment of the invasive region around the main tumor mass following tumor resection may have advantage in both efficacy and safety compared to low-LET-based therapies due to potential reduced radioresistance and the localized nature of the treatment, with minimal damage to adjacent tissue. Compared to targeted alpha therapy for GBM, delivered by intratumoral or intracavity injection, DaRT has the advantage of providing controlled geometric coverage of the target volume, using dedicated applicators (which are presently under development) for deploying multiple seeds through a single entry point in the skull. This can allow avoiding uncertainties associated with catheter-based delivery, where drug spillage to healthy regions may greatly reduce the efficacy of the treatment and increase the risk for brain tissue toxicity.

Improving the survival of GBM patients presents an enormous challenge and an unmet need that requires the addition of novel therapeutic strategies to the clinical toolbox. Overall, the results in this study demonstrate the potential benefit of alpha radiation-based local radiotherapy in combination with drugs routinely used for GBM treatment in clinical settings, and support testing DaRT as a therapeutic tool in clinical trials for patients with GBM.

## Data availability statement

TThe original contributions presented in the study are included in the article/supplementary material, further inquiries can be directed to the corresponding author/s at cooks@bgu.ac.il.

## Ethics statement

The animal study was reviewed and approved by The Israeli government and institution guidelines and regulations (Ethics approval 01-20-055, IL-80-10-2020E).

## Author contributions

All authors have read and approved this manuscript. VD, TC and LA designed and supervised the study. YN, VD, TC, LA and MV wrote the manuscript. YN, MV, IL, SD, LE and AS performed the experiments. LD performed SAS-related statistical analysis. LE and MD performed the autoradiography and leakage experiments and analysis. IK and MS planned the irradiation station, insertion applicator, and DaRT seeds. RD, YK and IK provided clinical insights into treatment planning. All authors contributed to the article and approved the submitted version.

## Funding

This study was funded by Alpha-Tau Medical.

## Acknowledgments

We would like to thank Mr. Shmuel Packer, Mrs. Yevgeniya Korotinsky, and Mr. Yadin Cohen for their valuable advice.

## Conflict of interest

IK, LD and YK are consultants of the Alpha Tau Medical. RBD is the Chief Medical Officer of Alpha Tau Medical; YN, AS, MV, VD, MS, SD, and IL are employees of Alpha Tau Medical. YK, IK, VD, YN, TC, RBD, IL, MV, SD, and AS are the authors of a patent application related to this study. TC, MS, LA, IK and YK are shareholders in Alpha Tau Medical. VD, MS and LD hold stock options of Alpha Tau Medical. LD is the CEO of Biostatistics Statistical Consulting. MD receives a scholarship from Alpha Tau Medical. Alpha Tau Medical is the major funder of this study. The entire experimental design and data analysis and interpretation were conducted by the academic faculty and the scientists of Alpha-TAU Medical with no intervention/influence/objection from any non-scientific entities. The remaining author declares that the research was conducted in the absence of any commercial or financial relationships that could be construed as a potential conflict of interest.

## Publisher’s note

All claims expressed in this article are solely those of the authors and do not necessarily represent those of their affiliated organizations, or those of the publisher, the editors and the reviewers. Any product that may be evaluated in this article, or claim that may be made by its manufacturer, is not guaranteed or endorsed by the publisher.

## Abbreviations

GBM, Glioblastoma multiforme; DaRT, Diffusing alpha-emitters radiation therapy; TMZ, Temozolomide; BEV, Bevacizumab; VEGF, Vascular endothelial growth factor; LET, Linear energy transfer.
